# NUAK1 governs centrosome replication in pancreatic cancer via MYPT1/PP1β and GSK3β‐dependent regulation of PLK4

**DOI:** 10.1002/1878-0261.13425

**Published:** 2023-04-03

**Authors:** Declan Whyte, George Skalka, Peter Walsh, Ania Wilczynska, Nikki R. Paul, Claire Mitchell, Colin Nixon, William Clarke, Martin Bushell, Jennifer P. Morton, Daniel J. Murphy, Nathiya Muthalagu

**Affiliations:** ^1^ School of Cancer Sciences University of Glasgow UK; ^2^ CRUK Beatson Institute Glasgow UK; ^3^ Present address: Indian Institute of Technology Madras India

**Keywords:** centrosome replication, genomic instability, GSK3β, MYPT1, NUAK1, PDAC

## Abstract

The AMP‐activated protein kinase (AMPK)‐related kinase NUAK1 (NUAK family SNF1‐like kinase 1) has emerged as a potential vulnerability in MYC‐dependent cancer but the biological roles of NUAK1 in different settings are poorly characterised, and the spectrum of cancer types that exhibit a requirement for NUAK1 is unknown. Unlike canonical oncogenes, *NUAK1* is rarely mutated in cancer and appears to function as an obligate facilitator rather than a cancer driver *per se*. Although numerous groups have developed small‐molecule NUAK inhibitors, the circumstances that would trigger their use and the unwanted toxicities that may arise as a consequence of on‐target activity are thus undetermined. Reasoning that MYC is a key effector of RAS pathway signalling and the GTPase KRAS is almost uniformly mutated in pancreatic ductal adenocarcinoma (PDAC), we investigated whether this cancer type exhibits a functional requirement for NUAK1. Here, we show that high *NUAK1* expression is associated with reduced overall survival in PDAC and that inhibition or depletion of NUAK1 suppresses growth of PDAC cells in culture. We identify a previously unknown role for NUAK1 in regulating accurate centrosome duplication and show that loss of NUAK1 triggers genomic instability. The latter activity is conserved in primary fibroblasts, raising the possibility of undesirable genotoxic effects of NUAK1 inhibition.

## Graphical abstract

NUAK1 governs centrosome replication by regulating PLK4 levels via PP1β/MYPT1 and GSK3β. Suppression of NUAK1 leads to hyperactive PP1β and GSK3β, suppressing PLK4 turnover and leading to excessive centriole replication and genomic instability.

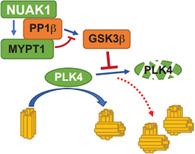

**Abbreviations**
4‐OHT: 4‐hydroxytamoxifen
AMPK: AMP‐activated protein kinase
APC: adenomatous polyposis coli
ARK: AMPK related kinase
CRC: colorectal cancer
DMEM: Dulbecco's modified eagle medium
EDTA: ethylenediaminetetraacetic acid
EGTA: ethylene glycol tetraacetic acid
EToH: ethanol
FBS: fetal bovine serum
GSK3β: glycogen synthase kinase 3 beta
IP: immunoprecipitation
KRAS: Kirsten rat sarcoma viral oncogene homologue
LATS1: large tumour suppressor kinase 1
LKB1: liver kinase B1
MEF: mouse embryonic fibroblast
MIP: maximum intensity projections
MTOC: microtubule organising centre
MYC: MYC proto‐oncogene
MYPT1: myosin phosphatase target subunit 1
NaCl: sodium chloride
NFκB: nuclear factor kappa B
NRF2: nuclear factor erythroid 2‐related factor 2
NUAK1: NUAK family SNF1‐like kinase 1
NUAK2: NUAK family kinase 2
PDAC: pancreatic ductal adenocarcinoma
PLK1: polo kinase kinase 1
PLK4: polo‐like kinase 4
PNUTS: protein phosphatase 1 (PP1) nuclear targeting subunit
PP1B: protein phosphatase 1 beta
PTEN: phosphatase and tensin homologue
RPMI: Roswell Park Memorial Institute
SIM: structured illumination microscopy
siRNA: short interfering RNA
TCGA: The Cancer Genome Atlas
TGFβ: transforming growth factor beta
TORC1: target of rapamycin complex 1
YAP1: yes‐associated protein 1

## 1 Introduction

Nuak1 is one of 13 AMPK‐related protein kinase (ARK) family members, all of which are activated upon phosphorylation by the tumour suppressor LKB1, encoded by *STK11* [1]. Diverse cellular activities have been ascribed to NUAK1, from roles in cell detachment and migration [2, 3, 4], governance of ploidy [5], regulation of mitochondrial trafficking [6], maintenance of mitochondrial fitness, function, and suppression of oxidative stress [7, 8, 9], to regulation of the spliceosome [10] and TORC1‐dependent protein translation [7, 11]. A full understanding of the mechanisms through which NUAK1 controls such diverse activities has, in many instances, proven elusive, and few reproducible targets of NUAK1 kinase activity have been identified to date [12]. Of these, the best‐characterised substrate for NUAK1 is the myosin phosphatase targeting subunit of the PP1β complex, MYPT1, encoded by *PPP1R12A*. MYPT1 is phosphorylated by NUAK1 on multiple serine residues, resulting in 14‐3‐3‐dependent attenuation of PP1β phosphatase activity, for example during cell detachment [2]. This activity of NUAK1 is shared by NUAK2, the closest related member of the ARK family. Importantly, this activity is unique amongst the ARKs, as NUAKs bind directly to the catalytic subunit of PP1β via their GILK motifs and appear to thus bind independently of their phospho‐target, MYPT1 [2]. Accordingly, another regulatory subunit of PP1β, PNUTS, encoded by *PPP1R10*, was also shown to be a substrate of NUAK1 during spliceosome formation [10].

Despite being activated by the tumour suppressor LKB1, a growing body of evidence indicates key roles for the NUAKs in cancer. We identified a requirement for NUAK1 to support ATP homeostasis upon acute elevation of MYC activity *in vitro* and for establishment of MYC‐driven hepatocellular tumours *in vivo* [7]. We subsequently demonstrated a similar requirement for NUAK1 to facilitate the NRF2‐dependent adaptation to oxidative stress in colorectal cancer, resulting in a targetable requirement for NUAK1 to maintain colonic tumours driven by loss of *Apc* and activating mutation of *KRas*, which converge to increase MYC expression [9]. Deletion of *NUAK1* or *STK11* (encoding LKB1) was each identified as synthetic lethal with *PTEN* loss in breast cancer [13], and loss of either NUAK1 or *STK11* drives a requirement for increased NFκB activity in ovarian cancer, again linked to suppression of oxidative stress [14]. High expression of *NUAK1* is associated with poor outcome in ovarian cancer where it promotes metastasis, in part via upregulation of fibronectin [15, 16]. Moreover, *NUAK1* mRNA is targeted by several microRNAs that reduce cancer cell migration and/or proliferation in a spectrum of cancer types (reviewed in [12, 17]). *NUAK2* is frequently amplified in breast and liver cancers [17], where the protein activates YAP1 transcriptional activity via inhibitory phosphorylation of the Hippo pathway kinase, LATS1, which negatively regulates YAP1 [18, 19]. NUAK2 was shown to be required for tumourigenesis in a YAP1‐driven model of hepatocellular carcinoma, while a dual‐specific NUAK inhibitor suppressed growth of YAP1 overexpressing tumour xenografts [18]. NUAK1 also inhibits LATS1 [5] and, akin to *NUAK2*, was recently described as a transcriptional target of YAP1/TEAD during TGFβ‐driven fibrosis [20], although the NUAKs are reported to play opposing roles in TGFβ signalling [21]. Importantly, both NUAKs retain kinase activity in LKB1‐deficient cancer cells, activated by an as‐yet undetermined mechanism [11].

Several groups have shown that MYC is a critical effector of mutant KRAS in pancreatic ductal adenocarcinoma (PDAC) [22, 23, 24, 25], prompting us to examine if NUAK1 might be required to support oncogenic proliferation in this context. Here, we reveal a requirement for NUAK1 to sustain proliferation of pancreatic cancer cells *in vitro* and identify a conserved role for NUAK1 in governing centrosome replication through GSK3β‐dependent control of PLK4 protein levels.

## 2 Materials and methods

### 2.1 Cell lines and cell culture

Mia PaCa‐2 (RRID:CVCL_0428) cells were obtained from ATCC and cultured in DMEM containing 2 mm l‐Glutamine, 100 μg·mL^−1^ of streptomycin, 100 μg·mL^−1^ of penicillin, 4.5 g·L^−1^ glucose, and 10% FBS. DAN‐G (RRID:CVCL_0243) were obtained from Professor Kevin Ryan and were cultured in RPMI containing 2 mm l‐glutamine, 100 μg·mL^−1^ of streptomycin, 100 μg·mL^−1^ of penicillin, 4.5 g·L^−1^ glucose and 10% FBS. All cell lines were tested periodically for mycoplasma. All cell lines were validated by short tandem repeat (STR) profiling by the Beatson Institute in‐house service (CRUK‐BICR). Primary mouse embryonic fibroblasts were generated as previously described [26]. To delete floxed *Nuak1*, MEFs were selected on Puromycin for 48 h, and CreER^T2^ was subsequently activated by treatment with 100 nm 4‐OHT. Cells in log phase growth were treated with the indicated concentrations of 5 or 10 μm of NUAK1 inhibitor (HTH‐01‐015, Tocris, Bristol, UK), GSK3 inhibitor 3 μm – (CHIR99021, Tocris), PLK4 inhibitor 100 nm (Centrinone, MedChem Express, Monmouth Junction, NJ, USA) throughout the study. Where indicated, cells were pre‐incubated with GSK3 inhibitor or PLK4 inhibitor for 1 h before a PBS wash and media change. Equivalent volumes of DMSO were used as vehicle controls. Cells in a 6 cm plate were lysed in 150 μL, cells in a 10 cm plate were lysed in 300 μL of Cell lysis buffer (150 mm NaCl, 50 mm Tris, pH 7.5, 1% NP‐40, 0.5% sodium deoxycholic acid, 1% SDS, plus complete protease and phosphatase inhibitor cocktail), then sonicated for 10 s at 40% amplitude using the Sonics Vibra Cell Sonicator (Newtown CT, USA).

### 2.2 Double thymidine block and flow cytometry

Thymidine was added to the cells at a concentration of 2, 4, or 6 mm and left for 12 h overnight. The cells were washed with PBS, and then, fresh medium was added 12 h later thymidine was added to the cells at a concentration of 2, 4, or 6 mm. Cell cycle profiles were analysed on the Attune Flow Cytometer. Mia PaCa‐2 cells were harvested then fixed in 80% cold ethanol and left at −20 °C overnight. Cells were stained with 15 μL of 1 mg·mL^−1^ propidium iodide, 1 μL of 10 mg·mL^−1^ RNase A (Invitrogen, Waltham, MA, USA) and 400 μL of PBS. Cells were left at 37 °C for an hour prior to analysis on the Attune Flow Cytometer. For pre‐treatments with GSK inhibitor or centrinone, cells were treated with the drugs for 1 h prior to a PBS wash and fresh medium was then added with the described concentration of small molecule inhibitors.

### 2.3 Cell number and confluency data

Mia PaCa‐2 and DAN‐G cells were plated at a seeding density of 50 000 cells respectively, per well of a 6‐well plate. The following day 20 nm of siRNA control and two siRNAs (Qiagen *NUAK1* siRNA 3 FlexiTube 00108402 or *NUAK1* siRNA 5 FlexiTube 02224572, Hilden, Germany) targeting *NUAK1* were added and left for 48 h. Cells were then counted using the Casy Cell counter. To measure confluency, an IncuCyte™ (Sartorius, Göttingen, Germany) was used to monitor growth over time. Mia PaCa‐2 and DAN‐G cells were plated at a seeding density of 3000 cells per well of a 96‐well plate. The following day drugs were added in fresh medium and left for up to 96 h. Drugs were added at a 2× concentration due to the existing medium still being present from seeding (100 μL of new medium containing 2× concentration was added to 100 μL existing medium to give 1× drug concentration).

### 2.4 Cell death analysis

Cells were seeded at a density of 1 × 10^5^ in a 6‐well plate and left to settle overnight. Ten micromolar HTH‐01‐015 or DMSO were added with the appropriate controls and left for 24 h in the Mia PaCa‐2 cells or 48 h in the DAN‐G cells. The supernatant was collected into 5 mL round bottom FACS tubes. Five hundred microliter of PBS is used to wash the cells and collected into the tube, and trypsin was then added to lift all the cells from the well. The trypsin was quenched with 100 μL of FBS and the cells were spun at 300 x *g* for 5 min. Cells were resuspended in 200 μL Annexin V binding buffer (10 mm Hepes pH 7.4, 140 mm NaCl, 2.5 CaCl_2_) containing 2 μL of Alexa Flour 647 Annexin V (BioLegend 640912, San Diego, CA, USA). The cells were left in the dark for 10 min at room temperature. Prior to analysis on the Attune FACS machine, 100 μL of 1 mg·mL^−1^ propidium iodide solution (Sigma‐Aldrich P4170, St Louis, MO, USA) was added. Each experiment had three technical replicates and repeated at least three times independently.

### 2.5 Plasmid transfection

GSK3β expression plasmids, previously described in [27, 28], were provided by Prof. Jim Norman (CRUK Beatson Institute). pCMV5 was transfected as an empty vector control. One microgram of pCMV5, GSK3β S9A, and GSK3β K85R was added to 250 μL OPTI‐MEM. In a separate Eppendorf, 3 μL Lipofectamine 3000 and 1 μL p300 was added to 250 μL OPTI‐MEM (Thermo, Waltham, MA, USA). Following an initial incubation, the plasmid suspension was transferred into the tube containing Lipofectamine 3000 and p300 and left to equilibrate for 15 min. This master mix was then added in a dropwise manner onto 6 cm dishes with a cell confluency of 2 × 10^5^. Transfection was performed in pen/strep‐free medium and changed after 6 h, and Mia PaCa‐2 cells left for longer than 6 h showed toxicity and cell death. Cells were harvested 24 h later for immunoblotting.

### 2.6 Immunofluorescence and centrosome quantification/multinucleation

MiaPaCa‐2 cells were plated on coverslips in a 6 cm dish (2 × 10^5^ per well) and left overnight before treatments were added. Cells were harvested and washed in PBS 3× then fixed in 3.7% formalin (Thermo Fisher Scientific) for 5 min at room temperature. Coverslips were then washed in PBS 3× and permeabilised with 0.1% Triton X‐100 for 5 min at room temperature then washed in PBS 3×. Cells were blocked in 1% bovine serum albumin (BSA) for 1 h at room temperature then antibodies were added in the 1% BSA overnight at 4 °C: NUAK1, (CST 4458, 1 : 100, Danvers, MA, USA); γ‐tubulin, (Sigma T6557 1 : 100); α‐tubulin (CST 8058S, 1 : 100); pericentrin (Abcam 28144, 1 : 100, Cambridge, UK); DAPI 1 : 5000. Coverslips were washed before addition of secondary antibody at a concentration of 1 : 250 in 1% BSA. Upon completion of secondary antibody (Invitrogen Alexa Flour‐488, Goat anti‐Rabbit A11034, or Alexa Flour 568, Goat anti‐Mouse A11004), the coverslips were washed 2× in PBS then 1× in water and left to dry for 30 min before addition of 10 μl DAPI containing mounting medium (Vectashield, Vector Labs, Newark, CA, USA). Slides were imaged using a Zeiss 710 confocal microscope using a 40×/1.30 NA EC‐Plan‐Neofluar Oil DIC M27 or a 63×/1.40 NA Plan‐Apochromat DIC M27 objective. Images were acquired using zen black software (Zeiss, Jena, Germany) and processed using fiji (image j, Opensource software). To measure the effect of RNAi mediated suppression of MYPT1, two siRNAs (Qiagen *MYPT1* siRNA 5 FlexiTube 02628682 or siRNA 6 FlexiTube 03067806) were added for 24 h, and cells were harvested for analysis. Upon cell synchronisation by a double thymidine block, the Mia PaCa‐2 cells were left for 10.5 h and harvested for analysis in mitosis. To analyse the multinucleation the cells were harvested 13 h from release. Determination of cells in mitosis was assessed by chromosome condensation and clear centrosome poles. Mitotic cells were counted when they were clearly distinct from any neighbouring cells. Multinucleation was counted by counting at least 100 cells in randomised fields across the coverslip. To analyse the effect of post‐S phase inhibition of NUAK1, GSK3, or PLK4, Mia PaCa‐2 cells were double thymidine blocked, released in fresh medium for 6 h, and drugs were added at described concentrations for 4.5 h for centrosome analysis or 7 h for multinucleation analysis.

image j was used to determine the mean fluorescence intensity of the NUAK1 staining in the siRNA knockdown experiments. Cell masks were created using Gaussian Blur, the thresholding method was Otsu, particles were analysed per cell with a size limit set at 10‐Infinity. The average particle mean fluorescence was then averaged per cell and expressed as a fold change compared to SiControl.

### 2.7 Super‐resolution microscopy

Cells were prepared as above using High Precision Coverslips No. 1.5H (Marienfeld, Lauda‐Königshofen, Germany) and mounted with Prolong Glass (Thermo Fisher Scientific). Samples were imaged using a Zeiss Elyra 7 Lattice Structured Illumination Microscopy (SIM) dual‐camera microscope using a 63×/1.40 NA Plan Apochromat Oil DIC M27 objective. Z‐stacks were acquired using 13 phases and optimal Z‐sectioning in zen black software. Images were processed using SIM2 processing, and maximum intensity projections (MIPs) were generated.

### 2.8 Immunoblotting

Denaturing PAGE was conducted using standard protocols. Primary antibodies for western blot were used at the following dilutions – NUAK1 (CST 4458, 1 : 500); pMYPT1 (MRC S5087, 1 : 400), MYPT1 (CST 8574 1 : 1000), PLK4 (CST 71033, 1 : 500), pGSK S9 (CST 5558, 1 : 500), β‐Actin (Sigma‐Aldrich A5441, 1 : 5000), Vinculin (Abcam 129002, 1 : 1000). Secondary horseradish peroxidase‐conjugated antibodies were added at a concentration of 1 : 5000 in 5% Milk in TBST (HRP anti‐Mouse NA931V; HRP anti‐Rabbit NA934V, both GE Healthcare (Chicago, IL, USA) or HRP anti‐Sheep 31 480 Pierce, Waltham, MA, USA) and detected using chemiluminescence Clarity or Clarity Max ECL western blotting substrate (Bio‐Rad 1705061 or 1705062, Hercules, CA, USA).

### 2.9 Immunoprecipitation

Anti‐PLK4 (CST 71033) and Protein G Sepharose 4 fast flow beads (GE Healthcare, 17‐0618‐01) were conjugated for 3 h at 4 °C with constant agitation. Fourteen million cells were harvested by scraping in IP buffer (50 mm Tris–HCl pH 7.5, 1 mm EDTA pH 8, 1 mm EGTA pH 8, 150 mm NaCl, 1% (v/v) NP40, 270 mm Sucrose, 1% (v/v) Protease inhibitor cocktail (#P8340, Sigma‐Aldrich), 1% (v/v) Phosphatase inhibitor cocktail 2 (#P5726, Sigma‐Aldrich), 1% (v/v) Phosphatase inhibitor cocktail 3 (#P0044, Sigma‐Aldrich)). The lysate was incubated on ice for 10 min, before centrifugation (10 min, 4 °C, 3000 ***g***). In parallel, the conjugated beads were washed in lysis buffer and aliquoted. The lysate was added to washed beads and incubated (16 h, 4 °C) with constant agitation. The beads were washed three times with 10× bead volume IP buffer. Finally, 2× bead volume of Laemmli buffer (32.6 mm Tris–HCL pH 6.8, 3.5% (v/v) SDS, 10% (w/v) Sucrose, 0.01% (w/v) β‐mercaptoethanol) was added to beads prior to boiling at 95 °C for 5 min. Antibodies used for IP – PLK4 (Cell Signalling Technology, 71033S) and Normal Rabbit IgG (Cell Signalling Technology, 2729).

### 2.10 Next generation sequencing

TriZol RNA precipitations were preformed according to a modified manufacturer's protocol: after the isopropanol precipitation step, the RNA pellet was resuspended in 100 μL H_2_O 100 μL acid phenol: chloroform (Ambion, Life Technologies, Carlsbad, CA, USA) was added, samples were vortexed for 20 s and spun at 9500 x *g* for 10 min. The aqueous phase was then extracted with 100 μL of chloroform and then spun at 9500 x *g* for 3 min. The aqueous phase was then precipitated with 2 μL glycogen, 10 μL NaAc, and 300 μL EtOH, and these were then stored at −20 °C for 24–48 h. Pellets were washed with 500 μL 75% EtOH, spun again at 13 000 r.p.m. for 5 min. Pellets were allowed to air dry, then resuspended in 20 μL H_2_O (Milli‐Q). RNA concentrations were obtained using a NanoDrop One (Thermo Fisher Scientific). RNA integrity was assessed using RNA ScreenTape (Agilent Technologies, Santa Clara, CA, USA) according to the manufacturer's protocol using a 2200 TapeStation System (Agilent Technologies). A RIN value > 7 was deemed acceptable. cDNA was generated using TruSeq stranded mRNA library prep kit (Illumina, San Diego, CA, USA) followed by NextSeq500 High output 75 cycle sequencing.

### 2.11 RNAseq analysis

FASTQ files were aligned to the human genome (hg19) using tophat2 [29] and then transformed into raw count data using featurecounts [30]. Differential expression analysis was performed using deseq2 [31] or edger [32]. Pathway enrichment was assessed using the metacore (Clarivate) enrichment analysis workflow. Original data are available from Arrayexpress, E‐MTAB 6244.

### 2.12 Statistical analysis

All experiments were performed on at least three separate occasions (biological replicates), except where noted in the figure legends. Graphs represent quantification of biological replicates, except where noted in the figure legends. Statistical analysis was carried out using graphpad 9.0 software (San Diego, CA, USA). Unpaired *t*‐tests were used when comparing two datasets, as indicated in each figure legend. When more than two data sets were compared, the statistical test ANOVA was used with *Post hoc* Tukey's multiple comparison test. Statistical significance was determined by *P* value *P < 0.05; **P < 0.01; ***P < 0.001; ****P < 0.0001.

## 3 Results

### 3.1 NUAK1 is overexpressed in PDAC

**Fig. 1:**
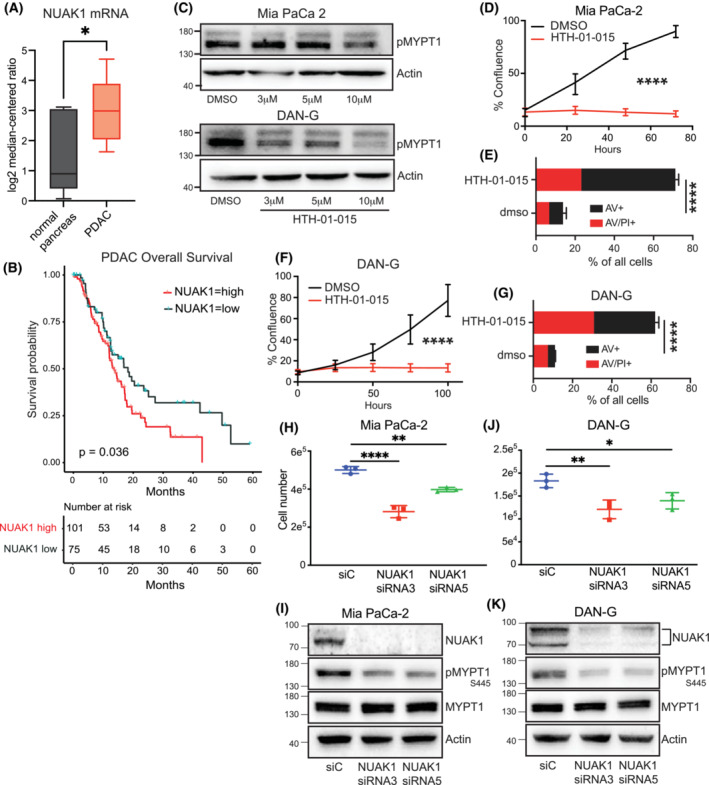
NUAK1 is overexpressed in Pancreatic Ductal Adenocarcinoma. (A) *NUAK1* mRNA levels in PDAC (*N* = 108) compared with expression in normal pancreas. Data from Badea et al. [33] analysed for *NUAK1*. *T*‐test used to determine statistical significance. Error bars represent standard deviation. (B) Kaplan–Meier plot of overall survival of PDAC patients with high versus low expression of *NUAK1*. Data from the TCGA cohort of PDAC. Logrank test. (C) Immunoblot of Ser445‐phosphorylated MYPT1 (pMYPT1) in lysates from Mia PaCa‐2 (upper panels and DAN‐G (lower panels) cells treated with the indicated concentrations of HTH‐01‐015 or DMSO vehicle. Representative blot of three independent experiments. (D) Confluence of Mia PaCa‐2 cells treated with 10 μm HTH‐01‐015 or DMSO vehicle, measured over time by Incucyte. Mean values ± SEM of three independent experiments shown. *T*‐test. (E) Cell death of Mia PaCa‐2 cells treated with 10 μm HTH‐01‐015 or DMSO vehicle for 24 h, measured by Annexin V/Propidium Iodide (PI) staining. Mean ± SEM of three independent experiments each having three technical replicates. **** denotes *P* < 0.0001 (*t*‐test). (F) Confluence of DAN‐G cells treated with 10 μm HTH‐01‐015 or DMSO vehicle, measured over time by Incucyte. Mean values ± SEM of three independent experiments shown. *T*‐test. (G) Cell death of DAN‐G cells treated with 10 μm HTH‐01‐015 or DMSO vehicle for 48 h, measured by Annexin V/Propidium Iodide (PI) staining. Mean ± SEM of three independent experiments each having three technical replicates. **** denotes *P* < 0.0001 (*t*‐test). (H) Cell number of Mia‐PaCa‐2 cells transfected with NUAK1 or non‐targeting (siC) siRNA, measured 48 h post‐knockdown measured using manual cell counting. Mean values ± SEM of three independent experiments shown. One‐way ANOVA with *post hoc* Tukey's test. (I) Immunoblot of pMYPT1 in lysates from Mia PaCa‐2 cells transfected with NUAK1 or non‐targeting (siC) siRNA. Representative blot of five independent experiments. (J) Cell number of DAN‐G cells transfected with NUAK1 or non‐targeting (siC) siRNA, measured 48 h post‐knockdown using cell counter. Mean values ± SEM of three independent experiments shown. One‐way ANOVA with *post hoc* Tukey's test. (K) Immunoblot of pMYPT1 in lysates from DAN‐G cells transfected with NUAK1 or non‐targeting (siC) siRNA. Representative blot of three independent experiments. For all panels, *P* value *P < 0.05; **P < 0.01; ****P < 0.0001.

Our previous work revealed increased expression of NUAK1 protein in a cohort of pancreatic ductal adenocarcinoma (PDAC) patient samples [7]. We therefore examined available datasets for expression of *NUAK1* mRNA. The Badea cohort of 108 PDAC cases [33] showed a pronounced increase in *NUAK1* mRNA compared with normal pancreas (Fig. 1A). Analysis of TCGA datasets via CBioPortal revealed PDAC has the highest average expression of *NUAK1* across multiple cancer types (Fig. S1A). We stratified the TCGA cohort of PDAC [34] into *NUAK1* high expression versus *NUAK1* low expression to determine if expression is associated with patient outcome and found significantly reduced survival of PDAC patients with high expression of *NUAK1* (Fig. 1B).

*NUAK1* expression in PDAC cell lines varies over a broad range (Fig. S1B). We selected DAN‐G and Mia PaCa‐2 cells as representative of *NUAK1* overexpression across the majority of PDAC lines. These PDAC lines were treated with a highly‐selective NUAK1 inhibitor, HTH‐01‐015, which lacks the off‐target activities of several other NUAK inhibitors [12, 18, 35]. Titration of HTH‐01‐015 in Mia PaCa‐2 and DAN‐G cells showed effective inhibition of NUAK1 kinase activity at 10 μm, as judged by MYPT1 phosphorylation on Ser445 (Fig. 1C and Fig. S1C), consistent with previous studies using the same antibody [9, 11, 35, 36]. Note that the residual p‐MYPT^S445^ signal in Mia PaCa‐2 cells likely reflects the redundant activity of NUAK2 on this site [11]. The same concentration of drug completely suppressed proliferation and induced abundant cell death in both cell lines when seeded at low density (Fig. 1D–G). To validate the on‐target effects of HTH‐01‐015, we used siRNA to specifically deplete NUAK1 and again found strong suppression of p‐MYPT^S445^ and significantly reduced proliferation in both cell lines, confirming the growth‐suppressive effects of the inhibitor data (Fig. 1H–K).

### 3.2 NUAK1 suppression drives nuclear defects *in vitro*

**Fig. 2:**
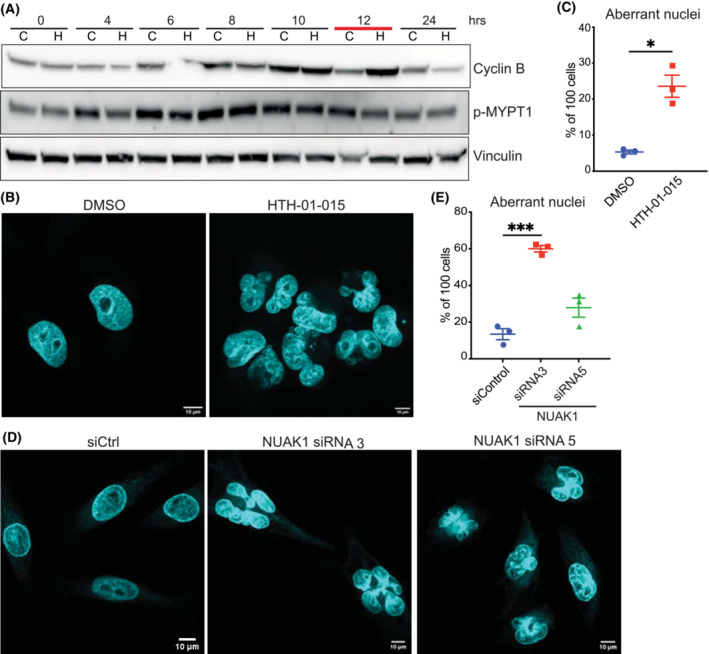
NUAK1 suppression drives nuclear defects *in vitro*. (A) Temporal analysis of Cyclin B expression in synchronised Mia PaCa‐2 cells treated with 10 μm HTH‐01‐015 (H) or DMSO vehicle (C) from time of release from thymidine block (0 h). Image is representative of three independent experiments. Red bar highlights delayed Cyclin B turnover upon NUAK1 inhibition, indicative of prolonged G2/M. Note that MYPT1 and Vinculin blots were performed on separate gels from the same lysates. (B) Representative images of DAPI stained, synchronised, Mia PaCa‐2 cells treated with 10 μm HTH‐01‐015 or DMSO vehicle from time of release. Cells were harvested for analysis 13 h after release from thymidine block, and at least 100 cells were counted per condition per experiment. Images are representative of three independent experiments. Scale bar = 10 μm. (C) Quantification of cells exhibiting nuclear defects following treatment with 10 μm HTH‐01‐015 or DMSO vehicle control as per (B). One hundred cells were scored per treatment group for each of three independent experiments. Mean ± SEM of three independent experiments shown. *T*‐test. (D) DAPI staining reveals nuclear defects in synchronised Mia PaCa‐2 cells transfected with NUAK1, or non‐targeting (siC), siRNAs. Cells transfected with NUAK1 si3 were fixed for analysis 24 h post‐transfection; cells transfected with NUAK1 si5 were fixed for analysis at 48 h port‐transfection. At least 100 cells were counted per condition per experiment. Images are representative of three independent experiments. Scale bar = 10 μm. (E) Quantification of 100 cells for each treatment group for each of three independent experiments, as per (D). One‐way ANOVA with *post hoc* Tukey's test. Mean ± SEM of three independent experiments shown. *T*‐test. For all panels, *P* value *P < 0.05; ***P < 0.001.

Examination of in‐house RNA‐SEQ datasets generated after inhibition (10 μm HTH‐01‐015) or shRNA‐mediated depletion of NUAK1 in U2OS cells revealed convergent enrichment for cell‐cycle (G1/S, S and G2/M signatures), DNA‐damage, and spindle microtubule‐related pathways (Fig. S2A,B). NUAK1 protein expression was previously shown to fluctuate with the cell cycle, peaking at entry to S‐phase, with reciprocal regulation of NUAK1 stability by Polo‐Like Kinase 1 (PLK1) and vice versa [36]. To determine if cell cycle defects contribute to suppressed proliferation of PDAC cells, we established a cell synchronisation protocol for Mia PaCa‐2 cells by double thymidine block and release. Upon release from thymidine block, Mia PaCa‐2 cells rapidly enter S‐phase, exiting S‐phase after 6 h, with the majority of cells in G2/M by 10 h post‐release (Fig. S2C,D). Treatment of Mia PaCa‐2 cells with HTH‐01‐015, starting from the time of release from thymidine block, resulted in delayed completion of G2/M, as measured by Cyclin B expression, and a sharp increase in cells exhibiting nuclear atypia, predominantly multinucleation with < 5% also exhibiting micronuclei, visible by DAPI staining (Fig. 2A–C). Depletion of NUAK1 by siRNA similarly increased the proportion of asynchronous Mia PaCa‐2 cells exhibiting multinucleation (Fig. 2D,E).

### 3.3 NUAK1 loss drives supernumerary centrosomes

**Fig. 3:**
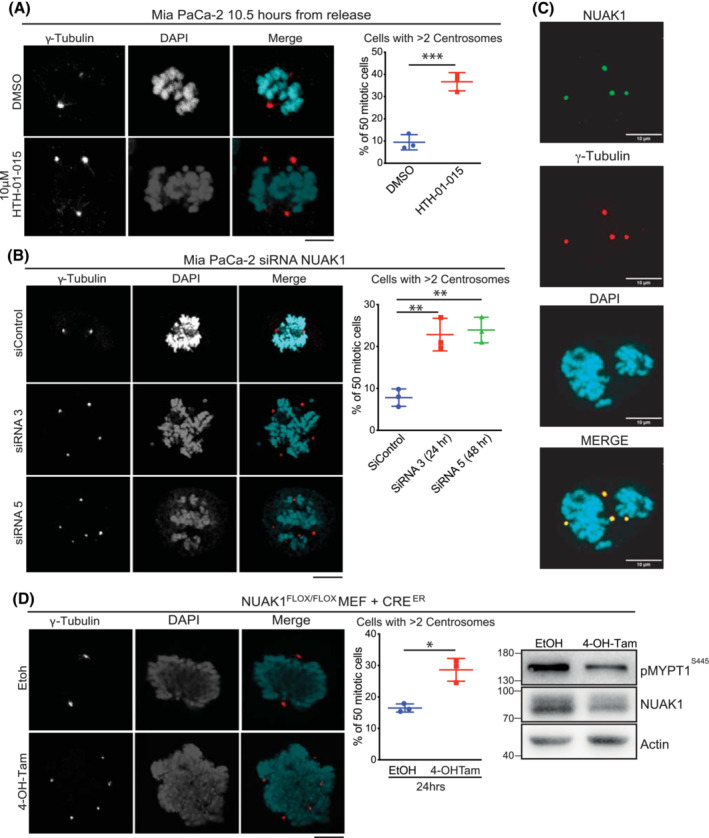
NUAK1 suppression drives ectopic centrosome replication. (A) Confocal analysis of γ‐tubulin immunofluorescence in synchronised Mia PaCa‐2 cells treated with 10 μm HTH‐01‐015 or vehicle control from time of release. Cells were fixed for analysis at 10.5 h post‐release from thymidine block. Right panel shows quantification of 50 mitotic cells per treatment group per experiment. Mean ± SEM of three independent experiments shown. *T*‐test. Scale bar = 10 μm. (B) Confocal analysis of γ‐tubulin IF in synchronised Mia PaCa‐2 cells transfected with NUAK1, or non‐targeting (siC), siRNAs. Cells transfected with NUAK1 si3 were analysed 24 h post‐transfection; cells transfected with NUAK1 si5 were analysed 48 h post‐transfection; both were analysed at 10.5 h from time of release from thymidine block. Graph shows quantification of 50 mitotic cells per treatment group per experiment. Mean ± SEM of three independent experiments shown. One‐way ANOVA with *post hoc* Tukey's test. Scale bar = 10 μm. (C) Representative images of confocal analysis of NUAK1 and γ‐tubulin IF staining in Mia PaCa‐2 cells at 10.5 h post‐release from thymidine block. Consistent results were obtained in three independent experiments. Scale bar = 10 μm. (D) Confocal analysis of γ‐tubulin staining in asynchronous *Nuak1* ^*FL/FL*^ MEFs, stably infected with retrovirus expressing Cre‐ER^T2^ and treated for 24 h with 4‐OH‐tamoxifen (to activate CreER^T2^) or EtOH vehicle. Graph shows quantification of 50 mitotic cells per treatment group per experiment. Mean ± SEM shown of three independent experiments shown. *T*‐test. Immunoblot of pMYPT1 in lysates from *Nuak1* ^*FL/FL*^ MEFs 24 h post‐4‐OH‐tamoxifen or EtOH vehicle treatment. Representative blot of three independent experiments. Scale bar = 10 μm. For all panels, *P* value *P < 0.05; **P < 0.01; ***P < 0.001.

The nuclear aberrations rapidly arising upon suppression of NUAK1 suggested a possible defect with chromosome segregation during mitosis. LKB1 has been shown previously to bind to centrosomes, which establish the spindle poles governing chromosome segregation. Loss of LKB1 was found to increase centrosome number, resulting in chromosome mis‐segregation and nuclear defects similar to those observed upon suppression of NUAK1 [37]. Mia PaCa‐2 cells were synchronised as above, treated with or without HTH‐01‐015, fixed at 10.5 h post‐release to capture mitosis, and stained for the centrosome marker γ‐tubulin. Confocal fluorescent microscopy revealed a sharp increase in mitotic cells with supernumerary (ie. > 2) centrosomes after treatment with HTH‐01‐015 (Fig. 3A). To validate this result, Mia PaCa‐2 cells were synchronised following transfection with *NUAK1* siRNA and harvested for γ‐tubulin staining during mitosis, 10.5 h post‐release. Depletion of NUAK1 with either of two siRNAs sharply increased the number of cells exhibiting supernumerary centrosomes, similar to treatment with the NUAK1 inhibitor (Fig. 3B). Immunofluorescent (IF) staining of untreated mitotic Mia PaCa‐2 cells for NUAK1 showed co‐localisation of NUAK1 and γ‐tubulin at the centrosomes (Fig. 3C and Fig. S3A) while time‐course analysis under the same conditions showed redistribution of NUAK1 over the cell cycle (Fig. S3B). IF staining for pericentrin showed similar overlap with NUAK1 as γ‐tubulin, confirming centrosomal co‐localisation (Fig. S3C). Consistent with the established roles for NUAK1 in regulating the cytoskeleton [2, 6], treatment with HTH‐01‐015 moreover resulted in pronounced disruption of the mitotic microtubule network (Fig. S3D).

To determine if the effect of NUAK1 suppression on centrosome number was cancer cell‐specific, we used floxed *Nuak1* ^*fl/fl*^ MEFs [38] stably expressing 4‐OH‐Tamoxifen‐inducible CRE recombinase fused to the modified ligand binding domain of the oestrogen receptor, CRE‐ER^T2^ [39]. Immunofluorescent γ‐tubulin staining showed significantly more cells exhibiting supernumerary centrosomes 24 h after activation of CreER^T2^ and deletion of *Nuak1*, compared with EtOH‐treated controls (Fig. 3D), showing that NUAK1 regulates centrosome number in untransformed fibroblasts as well as in cancer cells.

### 3.4 NUAK1 controls PLK4 protein levels

**Fig. 4:**
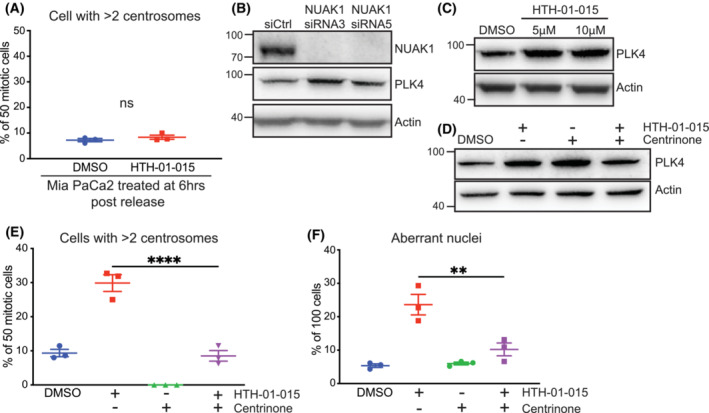
NUAK1 regulates PLK4 expression. (A) Quantification of centrosome number in 50 mitotic Mia PaCa‐2 cells per treatment group per experiment, measured by γ‐tubulin IF, fixed 10.5 h post‐release from thymidine block, and treated at 6 h post‐release with 10 μm HTH‐01‐015 or vehicle control. Mean ± SEM of three independent experiments shown. *T*‐test. (B) Immunoblot of PLK4 total protein in asynchronous Mia PaCa‐2 cells transfected with NUAK1, or non‐targeting (si Ctrl), siRNAs for 24 h. Representative of three independent experiments. (C) Immunoblot of PLK4 total protein in asynchronous Mia PaCa‐2 cells treated with the indicated concentrations of HTH‐01‐015 for 1 h. Representative of three independent experiments. (D) Immunoblot of PLK4 total protein in asynchronous Mia PacCa‐2 cells pre‐treated for 1 h with centrinone or vehicle, followed by 1 h treatment with HTH‐01‐015, vehicle, or HTH‐01‐015 + centrinone combined. Representative of three independent experiments. (E) Quantification of centrosome number by γ‐tubulin IF in mitotic Mia PaCa‐2 cells treated with Centrinone, HTH‐01‐015, both, or vehicle ctrl, from time of release from thymidine block. Cells were fixed for analysis at 10.5 h post‐release. Fifty cells were scored per treatment group per experiment. Mean ± SEM of three independent experiments shown. One‐way ANOVA with *post hoc* Tukey's test. (F) Quantification of incidence of nuclear aberrations in Mia PaCa‐2 cells treated with centrinone, HTH‐01‐015, both, or vehicle ctrl, from time of release from thymidine block. Cells were fixed for analysis at 13 h post‐release. One hundred cells were scored per treatment condition per experiment. Mean ± SEM of three independent experiments shown. One‐way ANOVA with *post hoc t*‐test. For all panels, *P* value; **P < 0.01; ****P < 0.0001.

Centrosome replication occurs early during S phase and is initiated by Polo‐like kinase 4 (PLK4) which is immediately targeted for proteasomal degradation, triggered by autophosphorylation, following duplication of the centrioles [40, 41]. To assess whether the inhibition during S‐phase is required for excessive centrosome replication, we treated synchronised cells with NUAK1 inhibitor after the completion of S‐phase. Treatment of synchronised Mia PaCa‐2 cells with HTH‐01‐015 following S‐phase, at 6 h post‐release, did not increase centrosome number, as measured by γ‐tubulin IF staining, consistent with a requirement for NUAK1 prior to completion of S‐phase (Fig. 4A). Overexpression of PLK4 is sufficient to drive excessive centriole replication resulting in supernumerary centrosomes [42]. Immunoblotting for PLK4 revealed increased protein expression upon depletion or inhibition of NUAK1 in both Mia PaCa‐2 (Fig. 4B,C) and DAN‐G cells (Fig. S4A,B). Co‐treatment of Mia PaCa‐2 cells with HTH‐01‐015 and the PLK4‐specific inhibitor centrinone [43] reversed the increased expression of PLK4 and suppressed both the supernumerary centrosome and aberrant nuclear phenotypes induced by NUAK1 inhibitor treatment from time of release from thymidine block (Fig. 4D–F).

### 3.5 NUAK1 regulates PLK4 via MYPT1/PP1β control of GSK3β activity

**Fig. 5:**
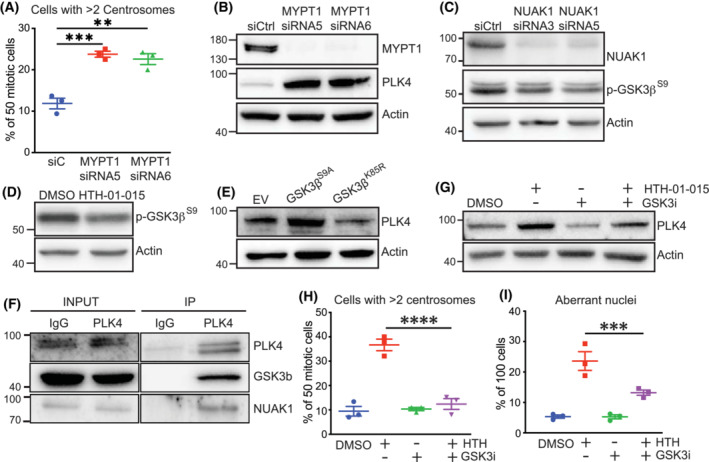
NUAK1 regulation of PLK4 is mediated by GSK3β. (A) Quantification of centrosome number by γ‐tubulin IF staining in synchronised Mia PaCa‐2 cells transfected with MYPT1, or non‐targeting (siC), siRNAs. Fifty cells scored per condition per experiment. Mean ± SEM of three independent experiments shown. One‐way ANOVA with *post hoc t*‐test. (B) Immunoblot of total PLK4 in Mia PaCa‐2 cells transfected with MYPT1, versus non‐targeting (siCtrl), siRNA. Representative of three independent experiments. (C) Immunoblot of Ser9 phospho‐GSK3β in Mia PaCa‐2 cells transfected with NUAK1, versus non‐targeting, siRNA. Representative of three independent experiments. (D) Immunoblot of Ser9 phospho‐GSK3β in Mia PaCa‐2 cells treated with 10 μm HTH‐01‐015 or DMSO vehicle for 4 h. Representative of three independent experiments. (E) Immunoblot of total PLK4 in Mia PaCa‐2 cells transiently transfected with constitutively active (S9A) or kinase‐dead (K85R) mutant GSK3β expression vector, compared with empty vector (EV). Representative of three independent experiments. (F) Immunoblots for endogenously expressed PLK4, NUAK1, and GSK3β in lysates and anti‐PLK4 immunoprecipitates, or IgG control IPs, of untreated asynchronous Mia PaCa‐2 cells. Representative of two independent experiments. (G) Immunoblot of total PLK4 in Mia PaCA‐2 cells pre‐treated for 1 h with 3 μm CHIR‐99021 (GSK3i) or DMSO vehicle, followed by 10 μm HTH‐01‐015 ± 3 μm CHIR‐99021 for 1 h immediately prior to harvest. Representative of three independent experiments. (H) Quantification of centrosome number by γ‐tubulin IF in mitotic Mia PaCa‐2 cells treated with CHIR‐99021 (GSK3i), HTH‐01‐015 (HTH), both, or vehicle ctrl, from time of release from thymidine block. Cells were fixed for analysis at 10.5 h post‐release. Fifty cells were scored per treatment condition per experiment. Mean ± SEM of three independent experiments shown. One‐way ANOVA with *post hoc* Tukey's test. (I) Quantification of incidence of nuclear aberrations in Mia PaCa‐2 cells treated with CHIR‐99021 (GSK3i), HTH‐01‐015 (HTH), both, or vehicle ctrl, from time of release from thymidine block. HTH‐01‐015 and ctrl values are the same as Fig. 4F as experiment was performed simultaneously. Cells were fixed for analysis at 13 h post‐release. One hundred cells were scored per treatment group per experiment. Mean ± SEM of three independent experiments shown. One‐way ANOVA with *post hoc* Tukey's test. For all panels, *P* value; **P < 0.01; ***P < 0.001; ****P < 0.0001.

The NUAK phospho‐target MYPT1 localises to centrosomes during mitosis, and roles for NUAK1 and MYPT1 in regulating PLK1 have been demonstrated [36, 44, 45]. We asked if MYPT1 might similarly regulate PLK4 expression. Indeed, depletion of MYPT1 in Mia PaCa‐2 cells, using either of two siRNAs, resulted in a profound increase in PLK4 protein expression and a sharp increase in the number of mitotic cells with supernumerary centrosomes (Fig. 5A,B). Our previously published analysis of HTH‐01‐015‐induced changes to the phospho‐proteome identified the inhibitory Ser9 phospho‐site of GSK3β as a NUAK1‐sensitive target of MYPT1/PP1β: treatment with HTH‐01‐015 reduced ROS‐dependent GSK3β^S9^ phosphorylation without impairing activity of the upstream kinase AKT, resulting in enhanced GSK3β kinase activity [9]. Phospho‐GSK3β^S9^ moreover localises specifically to the centrosomes during mitosis [46]. Suppression of NUAK1 by RNAi or HTH‐01‐015 reduced p‐GSK3β^S9^ in Mia PaCa‐2 cells (Fig. 5C,D), consistent with previous results from U2OS and CRC cell lines [9]. Overexpression of a constitutively active S9A GSK3β mutant increased, while a kinase‐dead K85R mutant decreased, expression of PLK4, consistent with a role for GSK3β in suppressing PLK4 turnover (Fig. 5E). Accordingly, the activating phosphorylation site Thr170 of PLK4 is predicted by recent phospho‐motif mapping [47] to be a perfect consensus for GSK3β kinase activity (Fig. S5). Both GSK3β and NUAK1 were moreover present in PLK4 immunoprecipitates (Fig. 5F). Treatment of Mia PaCa‐2 cells with the GSK3 inhibitor CHIR‐99021 [48] reduced expression of PLK4 accordingly and reversed the increase in PLK4 induced by NUAK1 inhibition (Fig. 5G). Co‐treatment of synchronised Mia PaCa‐2 cells with CHIR‐99021 and HTH‐01‐015 moreover suppressed the appearance of supernumerary centrosomes and significantly reduced the incidence of nuclear atypia induced by NUAK1 inhibition (Fig. 5H,I), establishing GSK3β as a critical mediator of NUAK1's role in regulating centrosome number via control of PLK4 protein levels.

## 4 Discussion

The orderly segregation of chromosomes during mitosis is vital to maintain genomic stability. This process is tightly choreographed as cells traverse the cell cycle, beginning in S‐phase with a single round of centriole duplication initiated by PLK4 activation [49]. Perturbations to PLK4 levels or activity suffice to disrupt the fidelity of centriole duplication, and simple overexpression of PLK4 drives excessive centriole replication resulting in supernumerary centrosomes and ultimately chromosome mis‐segregation at mitosis [42]. Our data identify a novel requirement for NUAK1 to ensure the fidelity of this process by controlling PLK4 protein levels. Nuak1 achieves this via phosphorylation of the PP1β regulatory subunit MYPT1, resulting in attenuated dephosphorylation of GSK3β at the inhibitory Serine 9 site. Loss or inhibition of NUAK1 thus results in hyperactive GSK3β and a consequent stabilisation of PLK4.

The rapid appearance of supernumerary centrosomes and consequent aberrant mitoses immediately following NUAK1 inhibition appears at first blush to be at odds with previous work suggesting that at least two rounds of mitosis are required following centrosome amplification for surplus centrosomes to result in chromosome mis‐segregation [50]. Indeed, we concur that regulation of PLK4 levels likely fails to alone account for the rapid appearance of aberrant nuclei in our study. Accordingly, PLK1 plays a vital role in centrosome disjunction and is also known to be regulated by NUAK1/MYPT1/PP1β [36, 51]. It is likely that PLK1 dysregulation following suppression of NUAK1 contributes to premature daughter centriole disjunction following PLK4‐induced over‐replication. Moreover, several laboratories have convincingly demonstrated PP1β hyperactivity following suppression of NUAK1 [2, 7, 9, 10, 36] and hyperactivation of PP1β is well‐documented to result in multiple mitotic defects [44, 52, 53, 54]. Furthermore, we provide clear evidence of the predicted loss of cell fitness and viability expected from such pleiotropic mitotic defects following NUAK1 inhibition [50]. The disentangling of the specific impact of NUAK1 suppression upon PLK4‐mediated centrosome replication from these pleiotropic effects of PP1β hyperactivity will thus require considerably more investigation.

A curious observation that emerged in the course of our work pertains to the normalisation of PLK4 levels and function upon co‐treatment with the PLK4 inhibitor Centrinone and the NUAK1 inhibitor HTH‐01‐015. PLK4 kinase activity is required for centrosome duplication but also triggers auto‐phosphorylation‐dependent degradation [41]. The increase in PLK4 levels detected upon Centrinone treatment alone is thus expected. The normalisation of PLK4 levels upon co‐treatment with Centrinone and HTH‐01‐015 is however rather perplexing and suggests that additional factors, conceivably including SCFβTRCP, involved in PLK1 regulation [36], participate in PLK4 turnover. We do however demonstrate that GSK3β physically binds to PLK4 and show that GSK3β activity is required for the stabilisation of PLK4 protein observed upon NUAK inhibition. The recently published phosphoproteome atlas [47] moreover predicts GSK3β as the kinase most likely to phosphorylate PLK4 within its activation T loop, adding further weight to our observations. Although this work does not address the precise mechanism of PLK4 stabilisation by GSK3β, our data do provide a mechanistic junction between several previously unlinked observations of LKB1, MYPT1, and Ser9‐phosphorylated GSK3β localisation to the centrosomes during mitosis [37, 44, 46, 55]. Accordingly, loss of LKB1 upstream and MYPT1 downstream of NUAK1 have each been shown to similarly result in genomic instability and nuclear aberrations of the nature described here [37, 55].

We find that a detectable fraction of cytosolic NUAK1 localises to the centrosome, most obviously visible during mitosis. In non‐mitotic cells, the centrosome governs additional sub‐cellular structures, such as the microtubule organising centre (MTOC) and the primary cilium [56, 57, 58]. Localisation to the MTOC is consistent with NUAK1's established role as a major regulator of the cytoskeleton [2] while possible participation in the primary cilium may suggest roles for NUAK1 in WNT and Hedgehog, amongst other, signalling pathways [56]. Interestingly, centrosomes are also present at the immune synapse where they govern delivery of secretory granules [59], suggesting a potential role for NUAK1 in cytotoxic immune cell effector function. This work thus opens new avenues for future investigation of NUAK1 in multiple new biological contexts.

Our analysis identifies a significant association of elevated NUAK1 expression with poor outcome in pancreatic ductal adenocarcinoma (PDAC). Treatment of PDAC cell lines with a highly selective NUAK1 inhibitor, HTH‐01‐015, was profoundly toxic, while NUAK1 depletion by RNAi significantly reduced proliferation of the same cell lines. These data concur with recent results from another lab using a distinct NUAK1 inhibitor [60] and with systems biology‐derived multi‐omic data showing a requirement for NUAK1 in PDAC [61]. Upregulation of NUAK1 moreover correlates with resistance of PDAC cells to Gemcitabine [62]. Moreover, PDAC is renowned for heavy deposition of fibrotic stroma [63] and NUAK1 was shown to mediate TGFβ‐induced fibrosis in multiple organs by enhancing YAP and TGFβ/SMAD signalling [20]. Although other work suggests an attenuating role for NUAK1 in TGFβ signalling [21], concurrent signalling, cell lineage, and organ context are all likely to influence this activity, and NUAK1 may thus participate in the pathology of PDAC via both tumour cell‐intrinsic and stromal activities. Teasing apart the specific roles of NUAK1 in PDAC will thus require generation of appropriately targeted animal models and development of small molecule inhibitors with both greater selectivity and *in vivo* potency. However, the suitability of NUAK1 as a potential therapeutic target in PDAC, or other cancers, must ultimately be weighed against the potential toxicity arising from the herein‐described requirement for NUAK1 to maintain genomic integrity through control of centrosome replication.

## 5 Conclusion

NUAK1 is required to maintain genomic stability by regulating accurate centrosome duplication during S‐phase. This activity of NUAK1 may restrict the clinical utility of NUAK1 inhibitors currently under development.

## Conflict of interest

DJM has received funding from the MERCK group for ongoing work not directly related to this manuscript. DJM has additionally received funding from Puma Biotech for unrelated work. MB has received funding from Celgene for unrelated work.

## Author contributions

DW, GS, DJM, and NM involved in conceptualization. DW involved in data curation. DW, GS, and PW involved in formal analysis. MB, JPM, and DJM involved in funding acquisition. DW involved in investigation. DW, CN, NRP, WC, and CM involved in methodology. DJM and NM involved in project administration. AW, JM, DJM, and NM involved in supervision. DW and GS involved in validation. DW involved in visualization. DW and DJM involved in writing (first draft). All authors involved in writing (review and editing).

### Peer review

The peer review history for this article is available at https://www.webofscience.com/api/gateway/wos/peer‐review/10.1002/1878‐0261.13425.

## Supporting information


**Fig. S1.** NUAK1 expression in PDAC (pertains to main Fig. 1).
**Fig. S2.** Transcriptomic impact of NUAK1 suppression (pertains to main Fig. 2).
**Fig. S3.** NUAK1 localisation to centrosomes (pertains to main Fig. 3).
**Fig. S4.** NUAK1 & GSK3β regulation of PLK4 protein levels (pertains to main Figs 4 and 5).
**Fig. S5.** Top 5 kinases predicted to phosphorylate Thr170 of PLK4.

## Data Availability

Original NGS data are available from Arrayexpress, E‐MTAB 6244.
