# Supplementary material for: NUAK1 governs centrosome replication in pancreatic cancer via MYPT1/PP1β and GSK3β‐dependent regulation of PLK4
Source: Mol Oncol. 2023 Apr 3;17(7):1212–27. doi: 10.1002/1878-0261.13425 (PMC10323901; doi:10.1002/1878-0261.13425)
Supplement: Supplementary file 1 — Fig. S1. NUAK1 expression in PDAC (pertains to main Fig. 1). Fig. S2. Transcriptomic impact of NUAK1 suppression (pertains to main Fig. 2). Fig. S3. NUAK1 localisation to centrosomes (pertains to main Fig. 3). Fig. S4. NUAK1 & GSK3β regulation of PLK4 protein levels (pertains to main Figs 4 and 5). Fig. S5. Top 5 kinases predicted to phosphorylate Thr170 of PLK4. [file MOL2-17-1212-s001.zip › Supplemental Figures S1-S5.pdf]

Figure S1

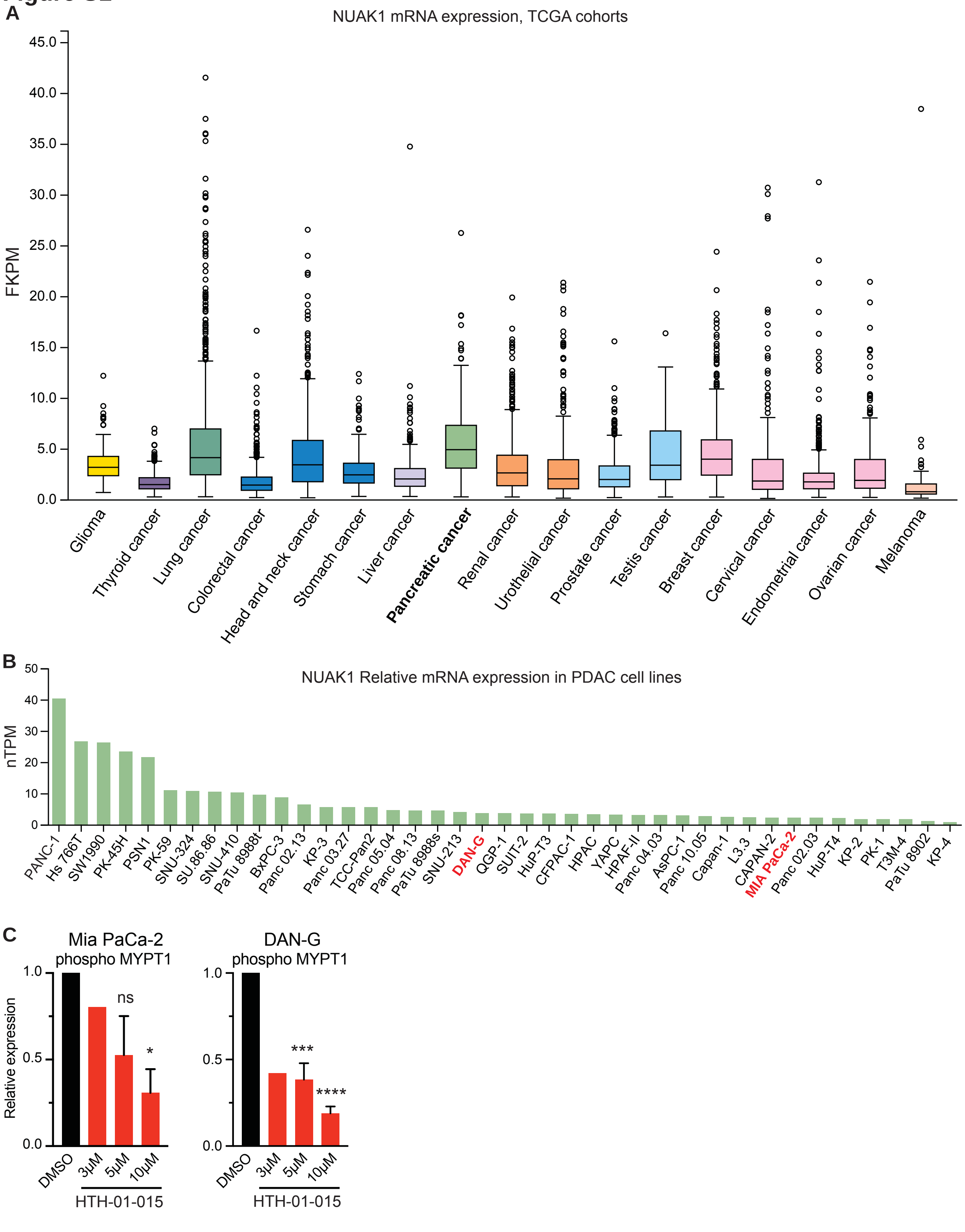
A
NUAK1 mRNA expression, TCGA cohorts
FKPM
45.0
40.0
35.0
30.0
25.0
20.0
15.0
10.0
5.0
0.0
Glioma
Thyroid cancer
Lung cancer
Colorectal cancer
Head and neck cancer
Stomach cancer
Liver cancer
Pancreatic cancer
Renal cancer
Urothelial cancer
Prostate cancer
Testis cancer
Breast cancer
Cervical cancer
Endometrial cancer
Ovarian cancer
Melanoma
B
NUAK1 Relative mRNA expression in PDAC cell lines
nTPM
50
40
30
20
10
0
PANC-1
Hs 766T
SW1990
PK-45H
PSN1
PK-59
SNU-324
SU.86.86
SNU-410
PaTu 8988t
BxPC-3
Panc 02.13
KP-3
Panc 03.27
TCC-Pan2
Panc 05.04
Panc 08.13
PaTu 8988s
SNU-213
DAN-G
QGP-1
SUIT-2
HuP-T3
CFPAC-1
HPAC
YAPC
HPAF-II
Panc 04.03
AsPC-1
Panc 10.05
Capan-1
L3.3
CAPAN-2
MIA PaCa-2
Panc 02.03
HuP-T4
KP-2
PK-1
T3M-4
PaTu 8902
KP-4
C
Mia PaCa-2
phospho MYPT1
Relative expression
1.0
0.5
0.0
ns
*
DMSO
3μM
5μM
10μM
HTH-01-015
DAN-G
phospho MYPT1
1.0
0.5
0.0
***
****
DMSO
3μM
5μM
10μM
HTH-01-015

Figure S2

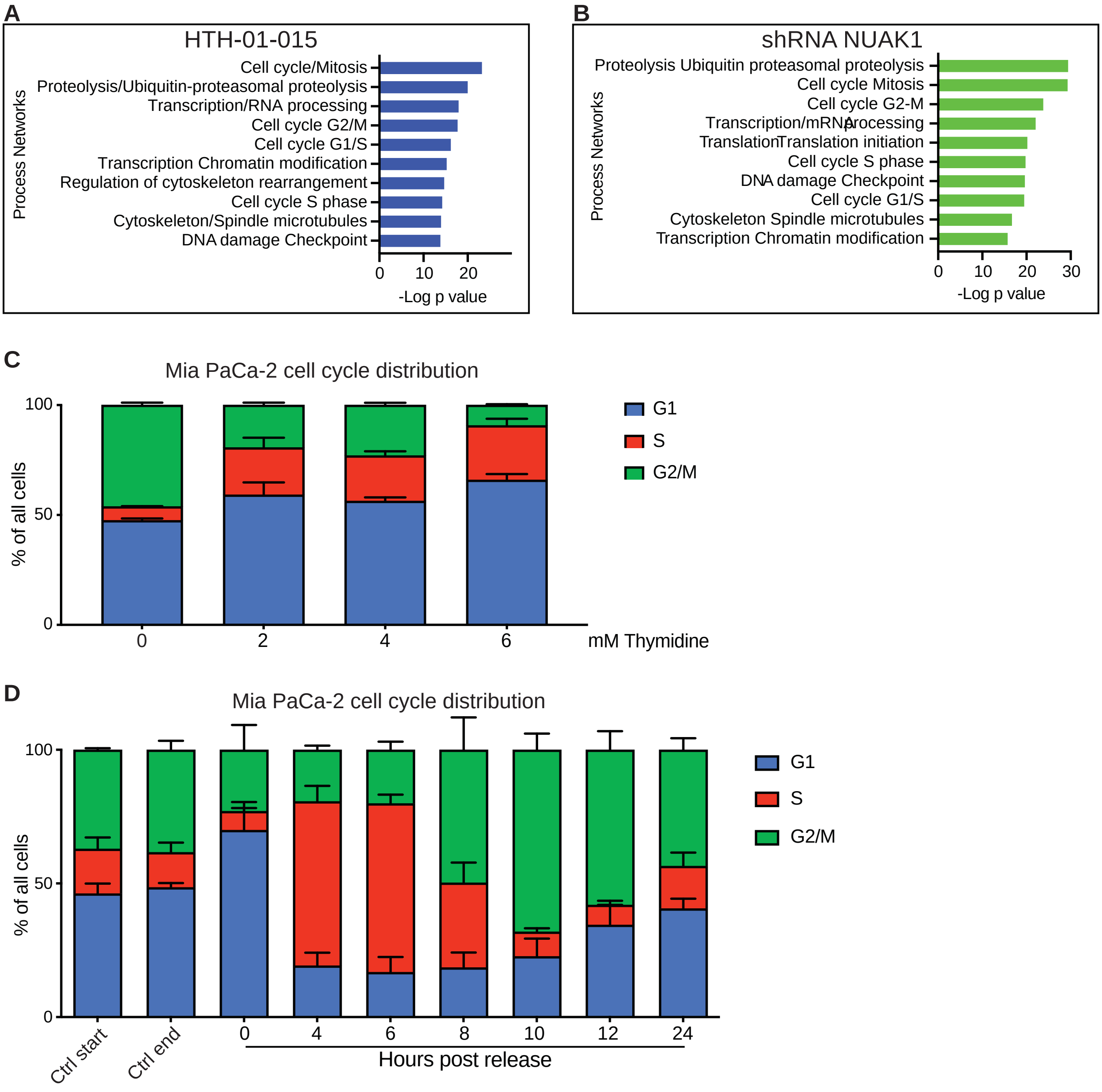

A
HTH-01-015
Process Networks
Cell cycle/Mitosis
Proteolysis/Ubiquitin-proteasomal proteolysis
Transcription/RNA processing
Cell cycle G2/M
Cell cycle G1/S
Transcription Chromatin modification
Regulation of cytoskeleton rearrangement
Cell cycle S phase
Cytoskeleton/Spindle microtubules
DNA damage Checkpoint
0 10 20
-Log p value
B
shRNA NUAK1
Process Networks
Proteolysis Ubiquitin proteasomal proteolysis
Cell cycle Mitosis
Cell cycle G2-M
Transcription/mRNA processing
TranslationTranslation initiation
Cell cycle S phase
DNA damage Checkpoint
Cell cycle G1/S
Cytoskeleton Spindle microtubules
Transcription Chromatin modification
0 10 20 30
-Log p value
C
Mia PaCa-2 cell cycle distribution
% of all cells
100
50
0
0 2 4 6
mM Thymidine
G1
S
G2/M
D
Mia PaCa-2 cell cycle distribution
% of all cells
100
50
0
Ctrl start
Ctrl end
0 4 6 8 10 12 24
Hours post release
G1
S
G2/M

Figure S3

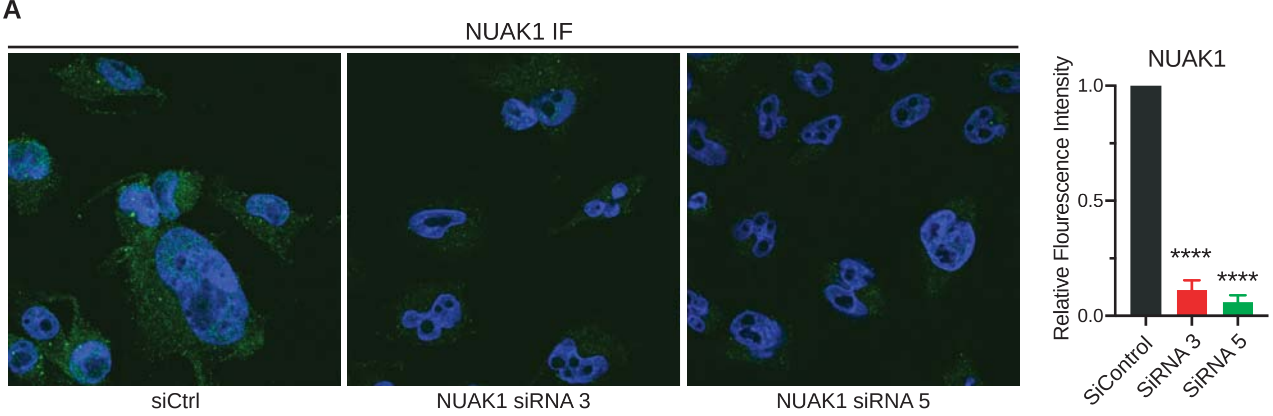
A
NUAK1 IF
siCtrl
NUAK1 siRNA 3
NUAK1 siRNA 5
NUAK1
Relative Flourescence Intensity
1.0
0.5
0.0
****
****
SiControl
SiRNA 3
SiRNA 5

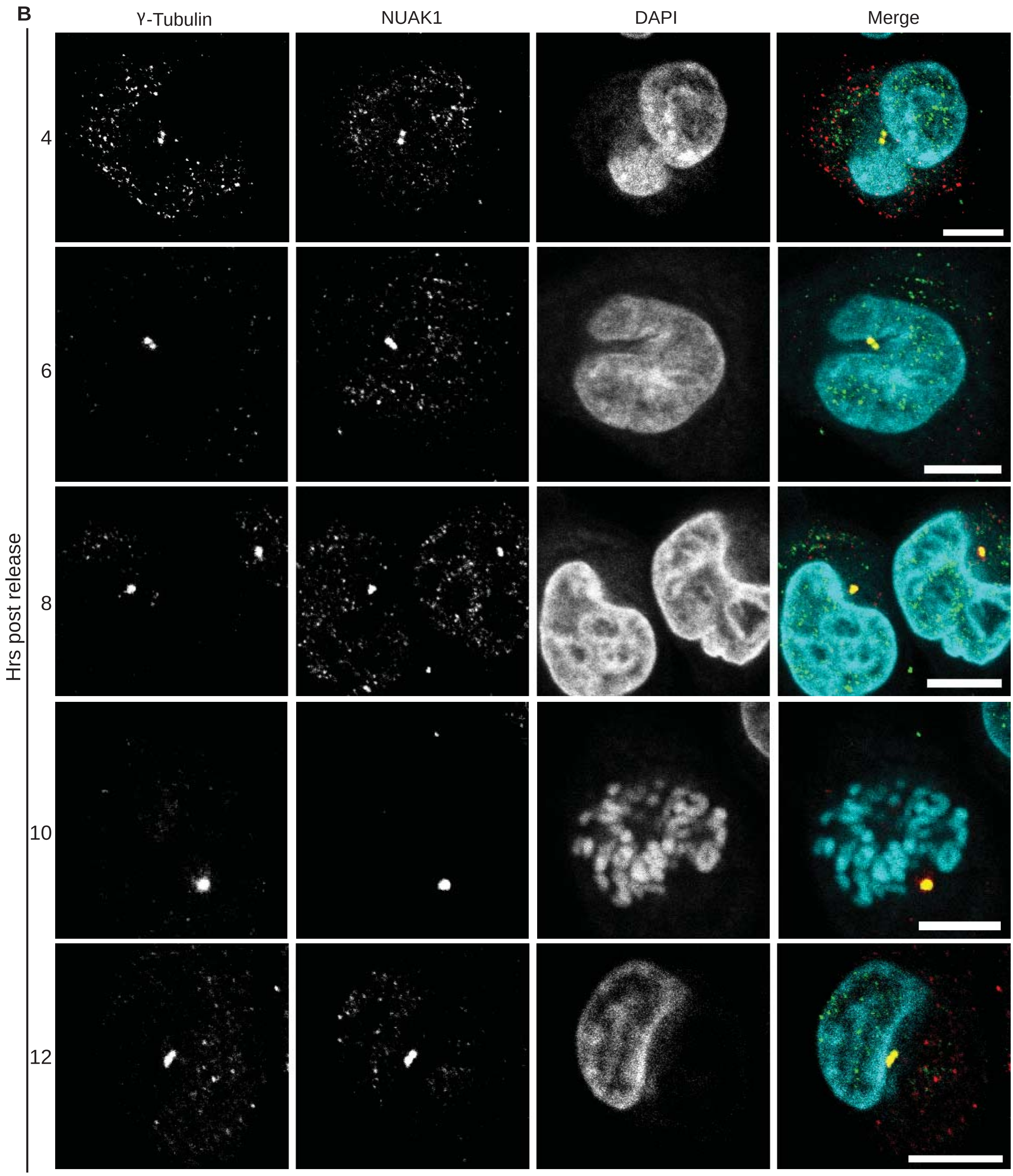
B
Ɣ-Tubulin
NUAK1
DAPI
Merge
Hrs post release
4
6
8
10
12

Figure S3C

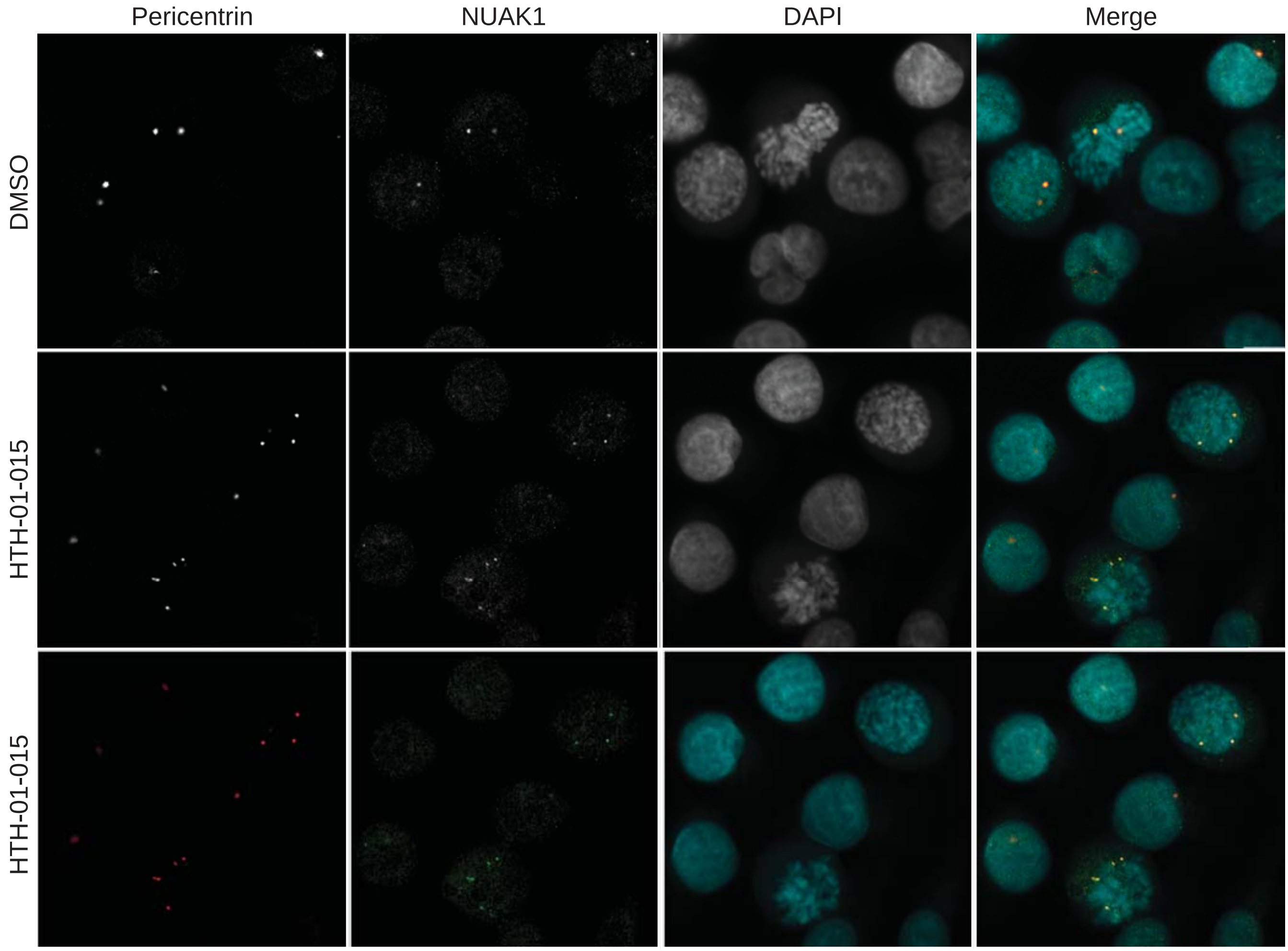

Figure S3D

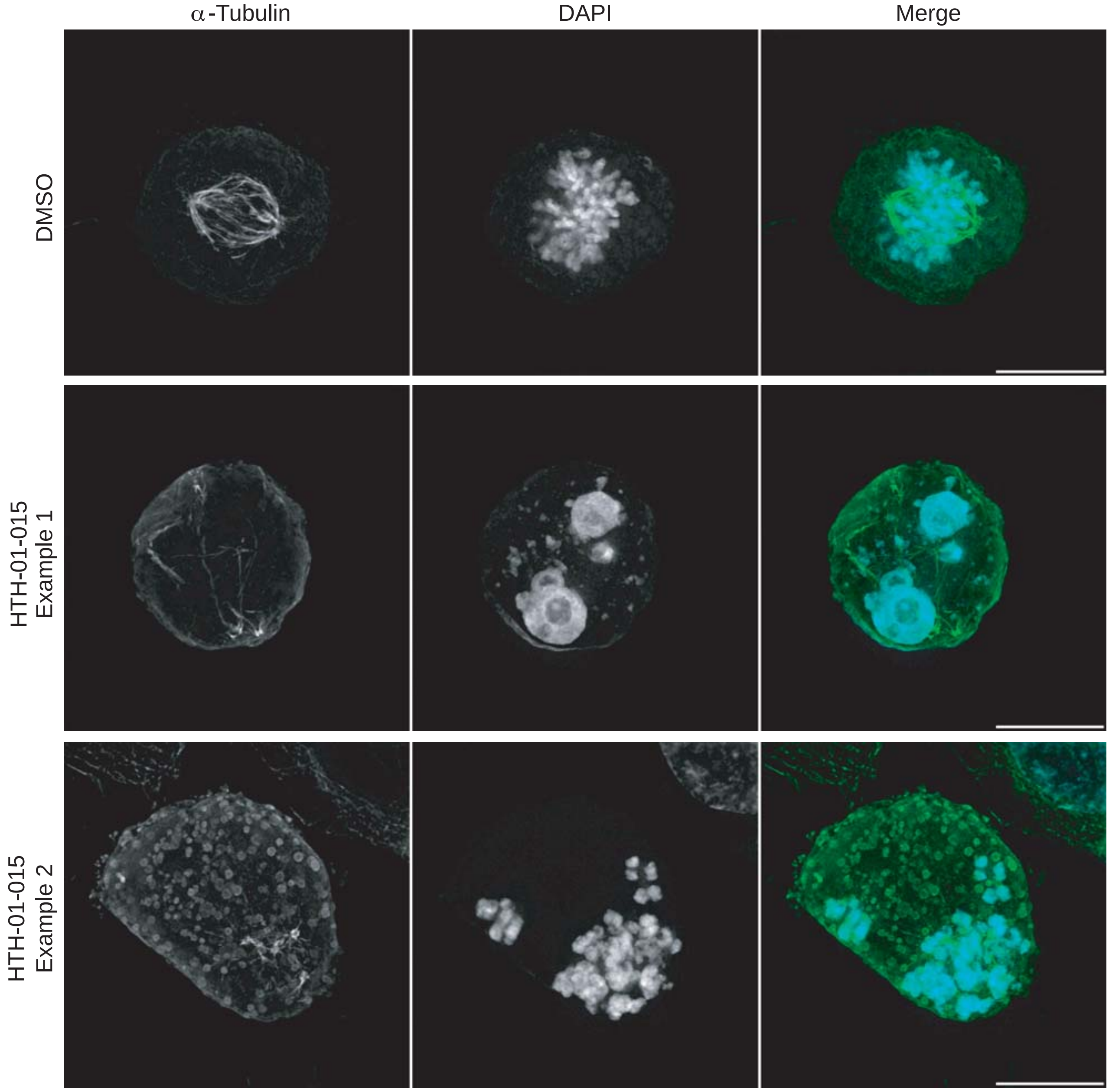
α-Tubulin
DAPI
Merge
DMSO
HTH-01-015
Example 1
HTH-01-015
Example 2

Figure S4

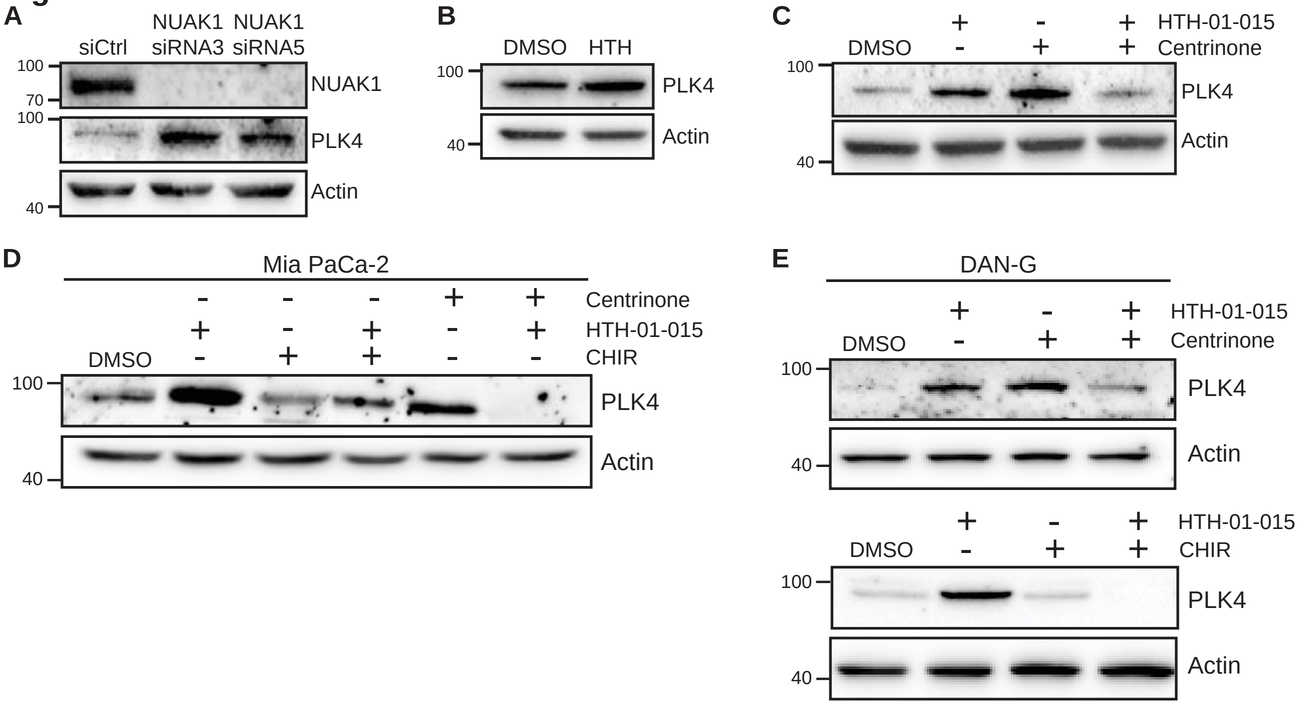
A
siCtrl
NUAK1 siRNA3
NUAK1 siRNA5
100
70
100
40
NUAK1
PLK4
Actin
B
DMSO
HTH
100
40
PLK4
Actin
C
DMSO
HTH-01-015
Centrinone
100
40
PLK4
Actin
D
Mia PaCa-2
Centrinone
HTH-01-015
CHIR
DMSO
100
40
PLK4
Actin
E
DAN-G
HTH-01-015
Centrinone
DMSO
100
40
PLK4
Actin
HTH-01-015
CHIR
DMSO
100
40
PLK4
Actin

Figure S5

PLK4 Thr 170 Phosphosite Plus advanced report

The Kinase Library   Score site   Score sites   Enrichment analysis   Powered by PhosphoSitePlus®

Hide advanced options

☑ Include phosphorylated residues in the surrouding sequence (phospho-priming)

Phosphorylated residues are denoted using lower-case letters (s for phosphoserine, t for phosphothreonine, y for phosphotyrosine). Otherwise, lower-case letters will be capitalized and treated as unmodified residues.

☑ Include Ser vs Thr favorability for the central phospho-acceptor

If enabled, the score will consider the kinase favorability for serine vs threonine as the phospho-acceptor.

Site sequence

position:	-5	-4	-3	-2	-1	0	1	2	3	4	5	6
sequence:	H	E	aK	H	Y	S/T	L	C	G	pT	P	N

Show 50 entries   Search:

	kinase	kinase group	log_2(score)	site percentile	percentile rank
	GSK3B	CMGC	5.216	100.000 %	1
	MEK1 [!]	STE	1.816	99.895 %	2
	TAK1	TKL	2.388	99.889 %	3
	MEK2 [!]	STE	1.333	99.888 %	4
	TAO3	STE	3.012	99.720 %	5
