# Supplementary material for: NUAK1 governs centrosome replication in pancreatic cancer via MYPT1/PP1β and GSK3β‐dependent regulation of PLK4
Source: Mol Oncol. 2023 Apr 3;17(7):1212–27. doi: 10.1002/1878-0261.13425 (PMC10323901; doi:10.1002/1878-0261.13425)
Supplement: Supplementary file 1 — Fig. S1. NUAK1 expression in PDAC (pertains to main Fig. 1). Fig. S2. Transcriptomic impact of NUAK1 suppression (pertains to main Fig. 2). Fig. S3. NUAK1 localisation to centrosomes (pertains to main Fig. 3). Fig. S4. NUAK1 & GSK3β regulation of PLK4 protein levels (pertains to main Figs 4 and 5). Fig. S5. Top 5 kinases predicted to phosphorylate Thr170 of PLK4. [file MOL2-17-1212-s001.zip › Supplementary Figure Legends.docx]

**Supplementary Figure Legends**

**Figure S1 NUAK1 expression in PDAC (pertains to main Figure 1)**

1. Comparison of NUAK1 mRNA expression across the indicated TCGA cancer cohorts. Data accessed via the Human Protein Atlas [64]. FKPM = Fragments per Kilobase/Million reads.
2. Comparison of NUAK1 mRNA expression in the indicated PDAC cell lines. Data accessed via the Human Protein Atlas: <https://www.proteinatlas.org/ENSG00000074590-NUAK1> . nTPM = normalised transcripts per million_)_
3. Quantification of Ser4465 phosphorylated MYPT1 in lysates from Mia PaCa-2 (left panel) and DAN-G (right panel) cells treated with the indicated concentrations of HTH-01-015. Mean ± SEM from 3 independent experiments shown, except for 3μM (done once).

For all panels p value *, P < 0.05; **, P < 0.01; ***, P < 0.001, **** P < 0.0001.

**Figure S2 Transcriptomic impact of NUAK1 suppression (pertains to main Figure 2)**

1. Pathway analysis of RNA-SEQ data from U2OS cells treated for one hour with 10μM HTH-01-015 versus DMSO vehicle.
2. Pathway analysis of RNA-SEQ data from U2OS cells depleted of NUAK1 by shRNA (Arrayexpress E-MTAB 6244).
3. Cell cycle analysis of Mia PaCa-2 cells upon treatment of the indicated concentrations of Thymidine. Mean ± SEM from 3 technical replicates from 1 experiments shown
4. Cell cycle analysis of Mia PaCa-2 cells arrested by double thymidine block and harvested at the indicated times following thymidine wash-out. Mean ± SEM from 3 independent experiments shown

**Figure S3 NUAK1 localisation to centrosomes (pertains to main Figures 3)**

1. Confocal microscopy analysis of NUAK1 immunofluorescent staining in asynchronous Mia-PaCa-2 cells transfected with NUAK1, or non-targeting, siRNA. Right panel shows quantification of total field fluorescence intensity from 3 independent experiments. Mean ± SEM shown.
2. Time-course confocal analysis of NUAK1 and γ-Tubulin localisation in synchronised Mia PaCa-2 cells following release from Thymidine block.
3. Confocal Microscopy analysis of NUAK1 and Pericentrin localisation in synchronised Mia PaCa-2 cells treated with DMSO or 10μM HTH-012-015, analysed at 10.5hrs following release from Thymidine block. Results are representative of 2 independent experiments.
4. Super-resolution microscopy of α-Tubulin localisation in synchronised mitotic Mia PaCa-2 cells treated as per (C) above with DMSO or 10μM HTH-01-015.

**Figure S4 NUAK1 & GSK3β regulation of PLK4 protein levels (pertains to main Figures 4 & 5)**

1. Immunoblot of PLK4 total protein in asynchronous DAN-G cells transfected with NUAK1, or non-targeting (si Ctrl), siRNAs for 24hrs. Representative of 3 independent experiments.
2. Immunoblot of PLK4 total protein in asynchronous DAN-G cells treated with 10μM HTH-01-015 for 1hr. Representative of 3 independent experiments.
3. Immunoblot of PLK4 total protein in asynchronous DAN-G cells pre-treated for 1hr with Centrinone or vehicle, followed by 1hr treatment with HTH-01-015, vehicle, or HTH-01-015 + Centrinone combined. Representative of 3 independent experiments.
4. Biologically independent replicate PLK4 immunoblot of lysates from Mia PaCa-2 cells treated with the indicated drugs as per main Figure 4D and 5G.
5. Biologically independent replicate PLK4 immunoblot of lysates from DAN-G cells treated with the indicated drugs as per Supplemental Figure 4B & C.

**Figure S5 Top 5 kinases predicted to phosphorylate Thr170 of PLK4**

Screenshot of PhosphoSitePlus kinase prediction for Thr170 of PLK4, using the advanced option for phosphor-priming. Accessed on March 4^th^ 2023.

https://www.phosphosite.org/kinaseLibraryAction.action?siteId=79924.
